# A Promising IFN-Deficient System to Manufacture IFN-Sensitive Influenza Vaccine Virus

**DOI:** 10.3389/fcimb.2018.00127

**Published:** 2018-05-01

**Authors:** Can Chen, Wenhui Fan, Jing Li, Weinan Zheng, Shuang Zhang, Limin Yang, Di Liu, Wenjun Liu, Lei Sun

**Affiliations:** ^1^CAS Key Laboratory of Pathogenic Microbiology and Immunology, Institute of Microbiology, Chinese Academy of Sciences, Beijing, China; ^2^University of Chinese Academy of Sciences, Beijing, China; ^3^CAS Key Laboratory of Special Pathogens and Biosafety, Wuhan Institute of Virology, Chinese Academy of Sciences, Wuhan, China

**Keywords:** Influenza A virus, vero cell line, NS1, interferon, vaccine

## Abstract

Interferon (IFN)-sensitive and replication-incompetent influenza viruses are likely to be the alternatives to inactivated and attenuated virus vaccines. Some IFN-sensitive influenza vaccine candidates with modified non-structural protein 1 (NS1) are highly attenuated in IFN-competent hosts but induce robust antiviral immune responses. However, little research has been done on the manufacturability of these IFN-sensitive vaccine viruses. Here, RIG-I-knockout 293T cells were used to package the IFN-sensitive influenza A/WSN/33 (H1N1) virus expressing the mutant NS1 R38A/K41A. We found that the packaging efficiency of the NS1 R38A/K41A virus in RIG-I-knockout 293T cells was much higher than that in 293T cells. Moreover, the NS1 R38A/K41A virus almost lost its IFN antagonist activity and could no longer replicate in A549, MDCK, and Vero cells after 3–6 passages. This indicated that the replication of NS1 R38A/K41A virus is limited in conventional cells. Therefore, we further established a stable Vero cell line expressing the wild-type (WT) NS1 of the WSN virus, based on the Tet-On 3G system. The NS1 R38A/K41A virus was able to steadily propagate in this IFN-deficient cell line for at least 20 passages. In a mouse model, the NS1 R38A/K41A virus showed more than a 4-log reduction in lung virus titers compared to the WT virus at 3 and 5 days post infection. Furthermore, we observed that the NS1 R38A/K41A virus triggered high-level of IFN-α/β production in lung tissues and was eliminated from the host in a relatively short period of time. Additionally, this virus induced high-titer neutralizing antibodies against the WT WSN, A/Puerto Rico/8/1934 (PR8), or A/California/04/2009 (CA04) viruses and provided 100% protection against the WT WSN virus. Thus, we found that the replication of the NS1 R38A/K41A virus was limited in IFN-competent cells and mice. We also presented a promising IFN-deficient system, involving a RIG-I-knockout 293T cell line to package the IFN-sensitive vaccine virus and a stable Vero cell line expressing NS1 to propagate the IFN-sensitive vaccine virus. The IFN-deficient system is applicable for the manufacture of IFN-sensitive vaccine virus.

## Introduction

Influenza A virus (IAV) causes seasonal epidemics and occasional pandemic infections in birds and mammals, including humans (Su et al., 2015, 2017). It poses a threat to global public health and causes economic losses. Effective influenza vaccines are urgently needed to prevent infections. Compared to killed inactivated and live attenuated influenza vaccines, interferon (IFN)-sensitive and replication-incompetent influenza viruses represent a revolution in vaccinology. These viruses are replication-incompetent in conventional cells but elicit robust humoral and cellular immune responses (Si et al., 2016; Du et al., 2018).

IAV is a member of the *Orthomyxoviridae* family with a genome containing an eight single-stranded negative RNA fragments (Portela and Digard, 2002; Samji, 2009), which encodes 14 viral proteins (Samji, 2009; Watanabe et al., 2010; Jagger et al., 2012; Wise et al., 2012; Muramoto et al., 2013). Non-structural protein 1 (NS1), which is one of the virulence factors of the virus and plays multiple roles during the life cycle of the virus, is encoded by the eighth fragment. It is well-known that NS1 acts as an IFN antagonist to antagonize RIG-I-mediated type I IFN production and support the replication of IAV. Furthermore, NS1 is capable of binding to viral RNA (Wang et al., 1999), RIG-I (Mibayashi et al., 2007), or the ubiquitin ligase TRIM25 (Gack et al., 2008, 2009; Rajsbaum et al., 2012) and Riplet (Rajsbaum et al., 2012), thereby, inhibiting the activity of IRF3 and NF-κB and the transcription of IFN (Talon et al., 2000a; Wang et al., 2000; Kuo et al., 2010). Additionally, NS1 also inhibits the maturation of pre-mRNA by binding to CPSF30 (Das et al., 2008; Ramos et al., 2013). One of the IFN-sensitive and replication-incompetent vaccine strains is the NS1-altered or -truncated influenza virus (Talon et al., 2000b; Donelan et al., 2003; Chambers et al., 2009; Wacheck et al., 2010; Pica et al., 2012). It is well established that the arginine 38 (R38) and lysine 41 (K41) residues of NS1 are the key sites for binding RNA and RIG-I to inhibit type I IFN production (Wang et al., 1999). Recombinant A/WSN/33 (WSN) and A/Puerto Rico/8/1934 (PR8) IAVs, expressing the mutant NS1 R38A/K41A protein, induce high levels of IFN-α/β and are attenuated in mice (Donelan et al., 2003; Gack et al., 2009; Ramos et al., 2013).

So far, studies have focused on the construction of IFN-sensitive and replication-incompetent vaccine viruses and the evaluation of their immunogenicity and protective efficiency. While little research has been done on the package efficiency and manufacturability of these IFN-sensitive viruses. In this study, we generated an IFN-sensitive and replication-limited recombinant WSN virus expressing the NS1 R38AK41A mutant in RIG-I-knockout 293T cells. The package efficiency of the NS1 R38A/K41A virus in RIG-I-knockout 293T cells was much higher than that in 293T cells. Moreover, we observed that the NS1 R38A/K41A virus could not survive in IFN-competent cells and mice for long periods of time because of the robust innate immune response. Thus, we further established a monkey kidney epithelial (Vero) cell line stably expressing WT NS1 of the WSN virus to propagate the IFN-sensitive virus.

## Materials and methods

### Cells and viruses

Madin-Darby canine kidney (MDCK), human alveolar epithelial (A549), Vero, human embryonic kidney (293T), and RIG-I-knockout 293T cells were maintained in Dulbecco's modified Eagle medium (DMEM) (Gibco) with 10% fetal bovine serum (FBS) (Gibco) at 37°C under 5% CO_2_ conditions. CRISPR/Cas9-based knockout of RIG-I in 293T cells has been previously described (Jiang et al., 2016). The IAV strain A/WSN/33 (H1N1) (WSN) was generated by using a 12-plasmid reverse genetics system (Zheng et al., 2015). The IAV strain A/Puerto Rico/8/1934 (H1N1) (PR8), IAV strain A/California/04/2009 (H1N1) (CA04), and Sendai virus (SeV) were propagated in the allantoic cavities of 9-day-old specific-pathogen-free embryonic chicken eggs (Merial, Beijing).

### Antibodies and reagents

Rabbit polyclonal antibodies against NS1 were obtained by immunizing animals with purified hexahistidine-tagged NS1 (His-NS1) (Zheng et al., 2017). Mouse monoclonal antibodies against M1 and rabbit polyclonal antibodies against NP were obtained as previously described (Koestler et al., 1984). Mouse anti-c-Myc (9E10) and mouse anti-FLAG (M2) antibodies were purchased from Santa Cruz Biotechnology, Inc. Rabbit anti-c-MYC antibodies and FLAG beads were purchased from Sigma. Rabbit anti-RIG-I (D14G6) was purchased from Cell Signaling Technology. Anti-GAPDH and anti-β-actin antibodies were purchased from Boao Rui Jing Technology Development Co., Ltd (Beijing). All secondary antibodies were obtained from Bai Hui Zhong Yuan Biotechnology. The lipofectamine reagent was purchased from Invitrogen. Opti-MEM was purchased from Gibco. The protease inhibitor cocktail was purchased from Roche. The Tet-On 3G Inducible Expression System was purchased from Clontech.

### Plasmid construction

The full-length WSN NS1 gene was cloned into the pCMV-MYC and pTRE3G vector. The full-length NS1 mutants (R38A, K41A, and R38A/K41A), either in pHH21 or pCMV-MYC, were generated with a Newpep site-directed mutagenesis kit (China) using the following primers: NS1-R38A-F, 5′-GACTTCTGATCTGCGCGAAGCCGAT-3′; NS1-R38A-R, 5′-ATTCCTTGATCGGCTTCGCGCAGAT-3′; NS1-K41A-F, 5′-TCCTCTTAGGGATGCCTGATCTCGG-3′; NS1-K41A-R, 5′-TTCGCCGAGATCAGGCATCCCTAAG-3′; NS1-R38A/K41A-F, 5′-CTCTTAGGGATGCCTGATCTGCGCG-3′; and NS1-R38A/K41A-R, 5′-TTCGCGCAGATCAGGCATCCCTAAG-3′. The full-length RIG-I gene was cloned into the pcDNA3-FLAG vector.

### Generation of recombinant IAVs

The IAV strain WSN and its NS1 mutants were generated using the 12-plasmid-based reverse genetics system. First, 293T and RIG-I-knockout 293T cells grown in 60-mm dishes to 80% confluency were transfected with 1 μg each of the 12 plasmids in the virus rescue system (NS1-lacking control groups represent the cells that were transfected with all the plasmids in the reverse genetics system, except for the NS1-expressing plasmid). Six hours later, the medium was replaced with DMEM containing 1 μg/mL tolylsulfonyl phenylalanyl chloromethyl ketone (TPCK)-treated trypsin. The cells were further cultured for 72 h at 37°C under 5% CO_2_ conditions, and the supernatants containing the rescued viruses were harvested and then centrifuged at 5,000 g for 5 min to remove cell debris.

### Plaque assays

Plaque assays to measure virus titers were performed as previously described (Zheng et al., 2017).

### RNA extraction, cDNA synthesis, and real-time quantitative PCR

Total RNA was extracted from cells using TRIzol (Ambion) according to the manufacturer′s instructions and the RNase-Free DNase Set (QIAGEN) was used to remove the residual DNA from the extracted RNA. Cellular RNA was transcribed into cDNA using a MLV reverse transcriptase kit (Promega) with oligo(dT) primers (TaKaRa). Quantitative real-time PCR assays were performed using SYBR Premix Ex Taq (TaKaRa). PCR was performed at 95°C for 30 s, followed by 40 cycles of 95°C for 5 s and 60°C for 31 s. The primers used for IFN, GAPDH, and NS1 were as follows: NS1-F, 5′-ATTCCGATGGATCCAAACACTGTGTCAAG−3′; NS1-R, 5′-CGAGATCAAGATTCTTCCTTCAGAATCC-3′; hIFN-β-F, 5′-TAGCACTGGCTGGAATGAGA-3′; hIFN-β-R, 5′-TCCTTGGCCTTCAGGTAATG-3′; mIFN-α-F, 5′-GGCTTGACACTCCTGGTACAAATGAG-3′; mIFN-α-R, 5′-CAGCACATTGGCAGAGGAAGACAG-3′; mIFN-β-F, 5′-GGAGATGACGGAGAAGATGC-3′; mIFN-β-R, 5′-CCCAGTGCTGGAGAAATTGT-3′; hGADPH-F, 5′-GGTGGTCTCCTCTGACTTCAAGA-3′; hGAPDH-R, 5′-GTTGCTGTAGCCAAATTCGTTGT-3′; mGAPDH-F, 5′-TTGTCTCCTGCGACTTCAACAG-3′; and mGAPDH-R, 5′-GGTCTGGGATGGAAATTGTGAG-3′. GAPDH served as the internal control. The expression of the target genes was normalized to that of GAPDH. The relative mRNA expression was calculated by the 2^−ΔΔCT^ formula (Livak and Schmittgen, 2001).

### Luciferase assay

The 293T cells were plated in 24-well plates and co-transfected with β-gal, NS1 (WT or mutant) proteins, and luciferase reporter plasmids (IFN-promoter-Luc). After 24 h, the cells were infected with SeV or PBS and harvested at 30 h post transfection (p.t.). The luciferase activity was detected using a Luciferase Assay Kit (Promega). The data were normalized to the activity of β-gal.

### Immunoprecipitation and western blot analysis

Cells were lysed for 40 min at 4°C using the lysis buffer (pH 7.4, 1%Triton X-100, 150 mM NaCl, 20 mM HEPES, 10% Glycerol, and 1 mM EDTA) supplemented with complete protease inhibitor cocktail following the transfection. Insoluble components were removed from lysates by centrifugation at 12,000 × *g* for 15 min. Lysates were incubated with anti-FLAG M2 affinity gel (Sigma) for 4 h. Following five washes with wash buffer (300 mM NaCl, 20 mM HEPES, 1% Triton X-100, 10% glycerol, and 1 mM EDTA), the precipitated proteins were separated by SDS-PAGE and then transferred onto PVDF membranes (Millipore Corporation, Billerica, MA). The membranes were blocked for 2 h in blocking buffer, and then probed with appropriate antibodies. Proteins were visualized using chemiluminescence detection reagents.

### Construction of the Tet-On 3G NS1 Vero cell line

The Tet-On 3G Inducible Expression System was used to establish the Tet-On 3G NS1-expressing cell line. Vero cells were transfected with the pCMV-Tet3G plasmid and selected by G418. G418-resistant clones with the highest fold induction by doxycycline (Dox) were maintained as the Tet3G-expressing Vero cell line. The Tet3G-expressing Vero cells were then transfected with pTRE3G-NS1 and a puromycin linear selection marker. Puromycin was used to select double-stable Tet-On 3G inducible cell lines. Individual double-stable clones were tested for the expression of NS1 in the presence of Dox, and clones with the highest fold induction were selected for further use.

### Virus infection

Female BALB/c mice (5-week-old, Vital River Laboratory, Beijing, China) were intranasally infected with 10^3^ plaque-forming units (PFU) of the rescued viruses or PBS. All mice were monitored daily for body weight and mortality for 14 days. Mice that lost 25% of their original body weight were pronounced dead. Three mice infected with different viruses or PBS were sacrificed at 12 h, 24 h, 3 days, 5 days, and 7 days post infection (p.i.), and then the lungs were collected. The lung index was calculated as 100% × (lung weight/body weight), and then lung tissue samples were homogenized in PBS or TRIzol to determine viral titers and relative gene expression. In addition, 50% mouse lethal dose (MLD_50_) values were determined by intranasally inoculating groups of five mice with 10-fold dilutions of WT virus, calculated by the Reed and Muench method (Biacchesi et al., 2005). Mice pre-inoculated with PBS or NS1 R38A/K41A virus in the previous experiments were infected with 10^3.88^ PFU of WT WSN virus at 17 d p.i. All mice were observed daily for clinical signs of disease, body weight and mortality. The animal research was approved by the Research Ethics Committee of Chinese Academy of Sciences, and complied with the Beijing Laboratory Animal Welfare and Ethical Guidelines of the Beijing Administration Committee of Laboratory Animals.

### Microneutralization (MN) assay

Serum samples were obtained from the blood of mice via centrifugation at 5,000 × *g* for 10 min. Before testing, serum samples were treated with receptor destroying enzyme (RDE, Denka Seiken, Japan). The MN assays were performed in MDCK cells by the diluted-serum constant-virus procedure (Edwards, 2006). Serum was first diluted in DMEM using the serial 2-fold dilution technique. Each dilution of serum (50 μL) was mixed with an equal amount of virus at 200 TCID_50_/0.1 mL and incubated for 1 h at 37°C. After incubation, the virus and serum mixtures were added to 96-well tissue culture plates containing confluent MDCK cell monolayers and incubated for 72 h at 37°C under 5% CO_2_ conditions. After the infection, cultures were monitored for the appearance of a cytopathic effect. Viral replication in the supernatants of each well was confirmed using the haemagglutination test.

## Results

### NS1 mutant IAVs were packaged in RIG-I-knockout 293T cells

**Figure 1:**
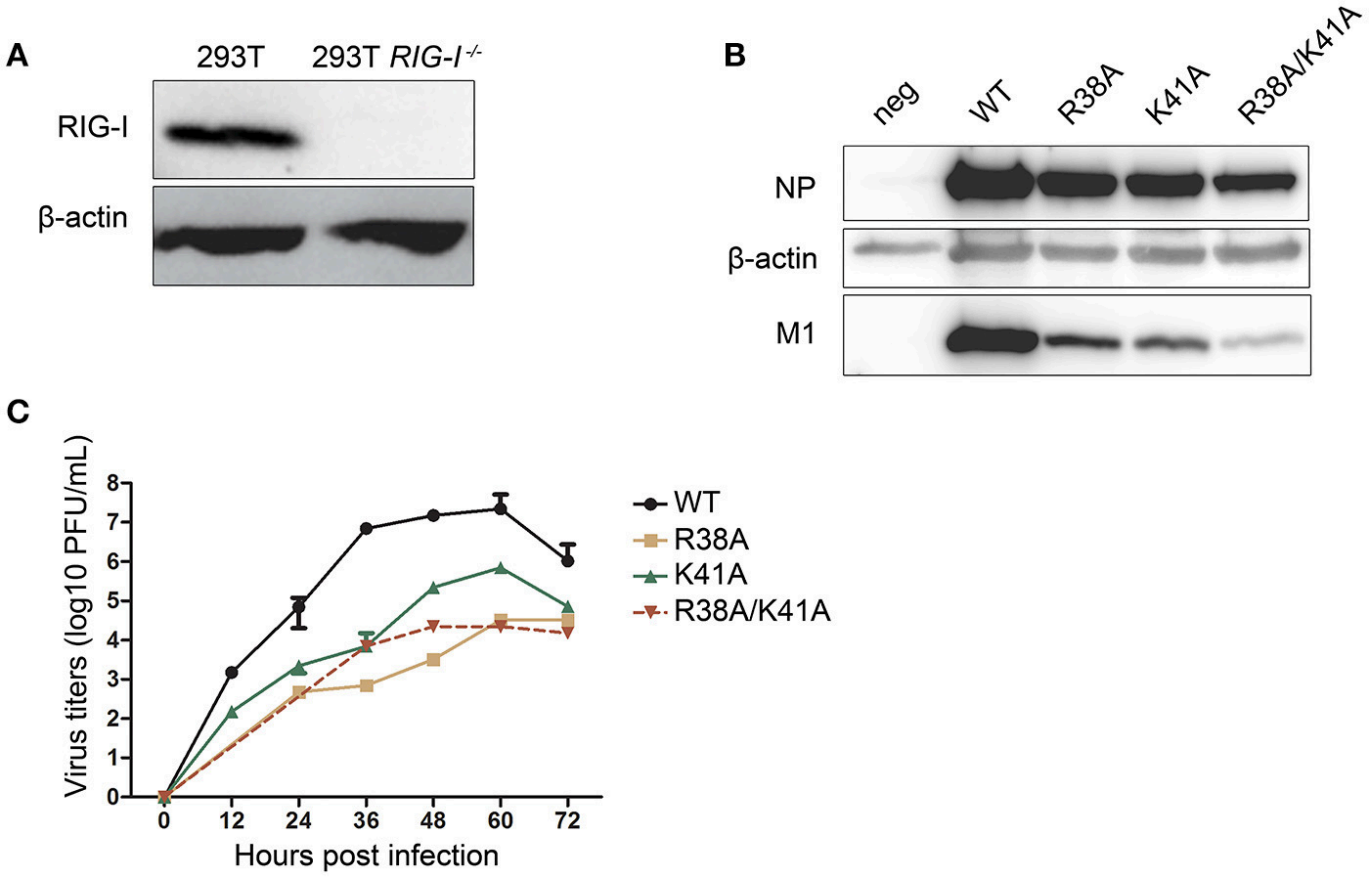
Construction of the NS1 mutant IAVs in RIG-I-knockout 293T cells. The wild-type (WT) and RIG-I-knockout 293T cells were infected with Sendai virus (SeV) for 12 h and lysed for western blot analysis **(A)**. The 12-plasmid reverse genetics system was used to rescue recombinant influenza viruses A/WSN/33 (WSN) expressing WT or mutant NS1 (R38A, K41A, or R38A/K41A) in 293T and RIG-I-knockout 293T cells. MDCK cells were then infected with the recombinant viruses rescued from the RIG-I-knockout 293T cells (MOI = 0.01) for 72 h and lysed for western blot analysis **(B)**. NP and M1 were detected with their respective antibodies, and β-actin was probed as the loading control. Virus titers **(C)** in supernatants from the infected MDCK cells were tested at the indicated time points. Growth curves were performed in triplicate. Error bars represented the standard deviations from the mean values of the three independent assays.

It has been reported that recombinant IAV expressing the mutant NS1 R38A/K41A induces high levels of type I IFN, which will, in turn, inhibit viral replication. We hypothesized that the knockout of RIG-I in 293T cells could facilitate the packaging of NS1 mutant recombinant IAVs by blocking the RIG-I-mediated type I IFN production signaling pathway. Therefore, we attempted to rescue NS1 mutant IAVs in WT and RIG-I-knockout 293T cells respectively. The absence of RIG-I in RIG-I-knockout 293T cells was examined by western blotting (Figure 1A). Alanine (A) substitution at R38, K41, or both (R38A, K41A, and R38A/K41A) was introduced in the pPolI-NS plasmid of the 12-plasmid reverse genetics system. These plasmids were then transfected into WT or RIG-I-knockout 293T cells as part of the reverse genetics system. At 72 h p.t., the supernatants were harvested. MDCK cells were infected with the harvested viruses for 72 h and lysed for western blot analysis. We found that the knockout of RIG-I in 293T cells enhanced the packaging efficiency of NS1 mutant recombinant IAVs. Additionally, the expression levels of NP and M1 in NS1 R38A/K41A virus-infected MDCK cells was much lower than those in NS1 R38A-, NS1 K41A-, and WT virus-infected cells (Figure 1B).

Next, we investigated the multiple-cycle growth kinetics of the rescued viruses. MDCK cells were infected with the WT and NS1 mutant viruses at a multiplicity of infection (MOI) of 0.001. The NS1 R38A and NS1 R38A/K41A viruses could not be detected until 24 and 36 h post infection (p.i.) respectively. The virus titers of the NS1 R38A and NS1 R38A/K41A viruses at each time point were reduced >1,000-fold compared to those of the WT virus, while the NS1 K41A virus showed replication efficiency intermediate between that of the WT and NS1 R38A/K41A viruses (Figure 1C).

**Figure 2:**
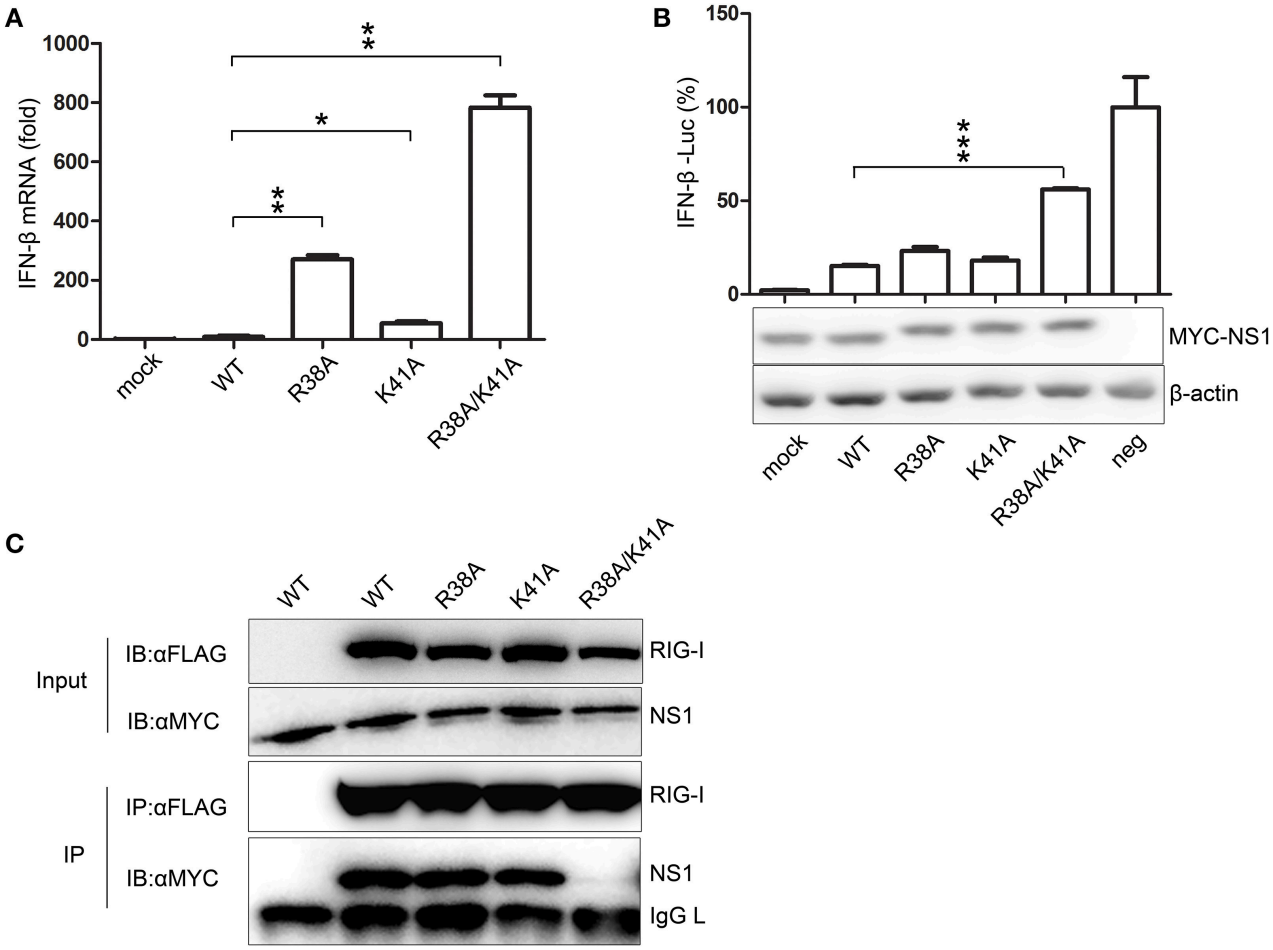
The NS1 R38A/K41A virus loses IFN antagonist activity. A549 cells were infected with WT, NS1 mutant viruses (MOI = 1), or PBS (mock) for 12 h. Real-time PCR **(A)** was performed to measure the production of IFN-β mRNA. Results are presented relative to mRNA level of IFN-β in WT virus-infected cells. All data were determined from three independent experiments. The differences between the WT and NS1 mutant viruses were analyzed by the Student's *t*-test. ^*^p < 0.05; ^**^p < 0.01; and ^***^p < 0.001. To measure the production of IFN-β with luciferase assays **(B**, upper**)**, 293T cells were transfected with the IFN-β-Luciferase reporter plasmid and MYC-NS1 WT, MYC-NS1 mutants or an empty vector. At 24 h p.t., cells were treated with SeV (MOI = 1) or PBS (mock) for 6 h. Results are presented relative to the luciferase activity in control cells transfected with the luciferase reporter and empty vector (neg). The differences between WT and NS1 mutants were analyzed by the Student's *t*-test. ^***^p < 0.001. The expression levels of NS1 and β-actin **(B**, lower**)** were detected by western blotting with specific antibodies. To determine the interactions between RIG-I and NS1 mutants **(C)**, 293T cells were transfected with FLAG-RIG-I plasmids and MYC-NS1 WT or MYC-NS1 mutants for 48 h. Anti-FLAG agarose beads were used to precipitate FLAG-RIG-I. MYC-NS1 WT or MYC-NS1 mutants were examined using anti-MYC antibodies.

To compare the effect of NS1 mutant viruses on the production of IFN-β, A549 cells were infected with WT or NS1 mutant viruses. The mRNA expression levels of IFN-β were quantified by real-time PCR. The NS1 R38A/K41A virus infection led to a remarkable up-regulation of IFN-β mRNA expression compared to the other viruses (Figure 2A). A luciferase system was also used to determine whether the mutant NS1 proteins were defective in reducing IFN-β production. The 293T cells were transfected with the IFN-β-Luciferase reporter plasmid and MYC-NS1 WT, MYC-NS1 mutants, or an empty vector. Sendai virus was added as an IFN-β stimulating factor. We observed that NS1 R38A/K41A recovered IFN production to the greatest extent compared to NS1 WT, NS1 R38A, and NS1 K41A (Figure 2B).

NS1 interacts with RIG-I, thus, inhibiting downstream signaling pathways and IFN production (Mibayashi et al., 2007; Jia et al., 2010). To determine whether the NS1 mutations play a role in the interaction between NS1 and RIG-I, we co-transfected MYC-NS1 WT or MYC-NS1 mutant expressing plasmids with FLAG-RIG-I into 293T cells for co-IP assays. The NS1 R38A/K41A mutant displayed remarkably decreased binding to RIG-I compared to NS1 WT, NS1 R38A, and NS1 K41A (Figure 2C). Thus, the interaction between the R38A/K41A mutant and RIG-I was hindered, resulting in more IFN production. All of these results suggested that the NS1 R38/K41 virus rescued in RIG-I-knockout 293T cells almost lost the IFN antagonist activity.

### Replication of NS1 R38A/K41A virus is limited in IFN-competent cells during successive passaging

**Figure 3:**
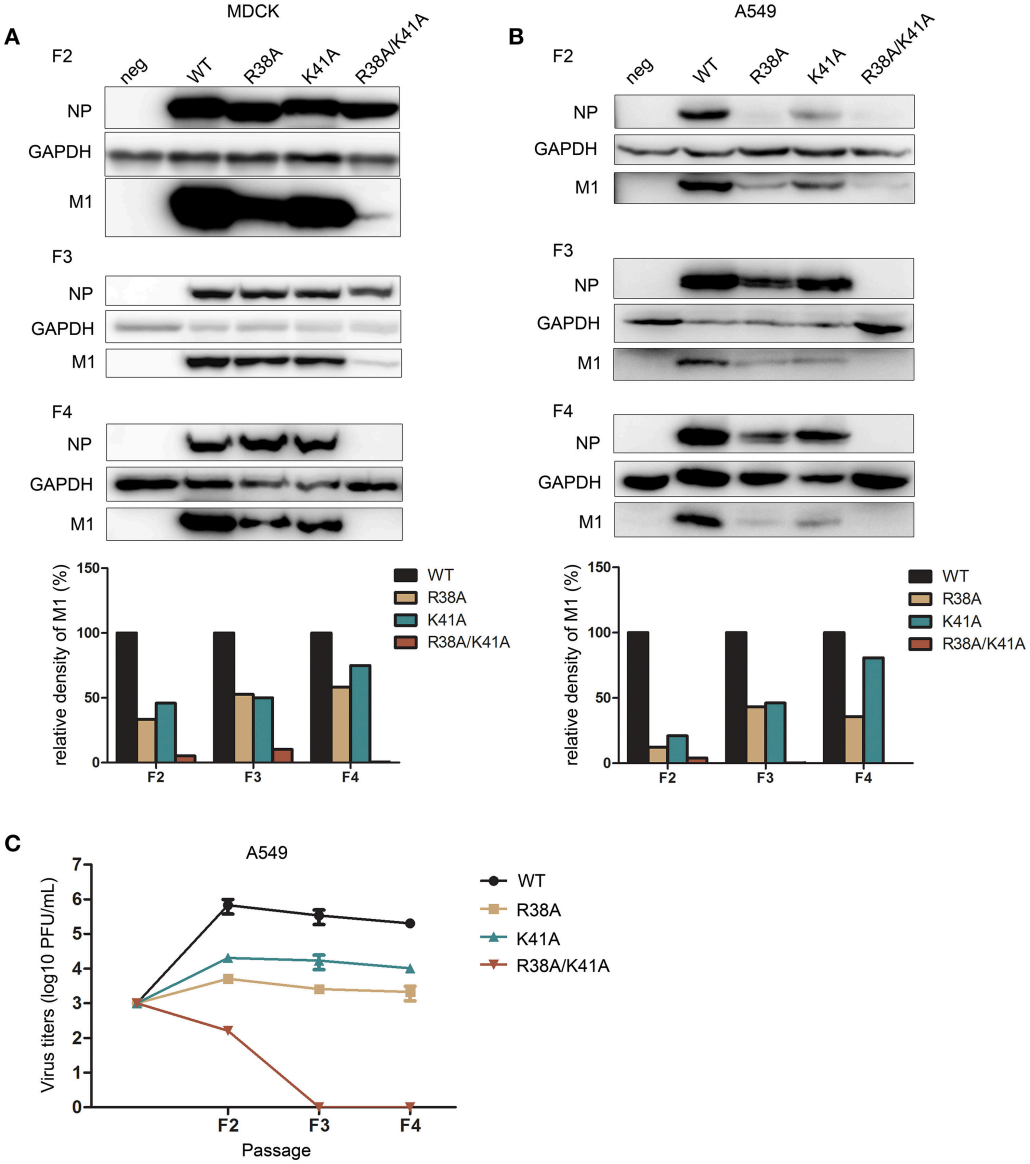
The replication of NS1 R38A/K41A virus is limited in IFN-competent cells during successive passaging. The rescued WT and NS1 mutant viruses of the first passage (F1) were blindly passaged in MDCK cells **(A)** till the fourth passage (F4). The same volume was used in all infections. The infected MDCK cells of each generation were lysed for western blot analysis. The expression levels of NP and M1 were detected with the respective antibodies (top). GAPDH was probed as the loading control. The relative expression levels of M1 were quantified (below). A549 cells **(B)** were infected with the rescued F1 WT or NS1 mutant viruses from MDCK cells at the same dose (MOI = 0.001). The harvested F2 viruses were blindly passaged to F4 in A549 cells. Virus from each passage was collected for western blot analysis. NP, M1, and GAPDH were detected (top). The relative expression levels of M1 were quantified (below). Virus titers **(C)** of F2 to F4 WT and NS1 mutant viruses in A549 cells were measured in MDCK cells by plaque assays.

Although many IFN antagonist activity-defective NS1-altered influenza viruses have been reported, most of these studies focused on the construction method and immunogenicity efficiency of the vaccine virus. Here, we paid more attention to evaluate the manufacturability of the NS1 mutant viruses in cells. Thus, we investigated the replication ability of NS1 mutant viruses in different cell lines during successive passaging. The rescued WT and NS1 mutant viruses of the first passage (F1) were passaged blindly in MDCK cells until the fourth passage (F4). The same volume of virus was used for all infections. Western blotting was performed to detect NP and M1 expression of MDCK cells in each passage. NP and M1 expression levels of the NS1 R38A/K41A virus were much lower than those of the WT and other NS1 mutant viruses, and the viral proteins of the NS1 R38A/K41A virus were not detected after 4 passages (Figure 3A), indicating that the replication ability of NS1 R38A/K41A virus was limited in MDCK cells. Next, we further determined the replication ability of the NS1 R38A/K41A virus in A549 cells with the same initial infectious dose. A549 cells were infected with the rescued viruses of F1 at the same dose (MOI = 0.001). We observed that the NP and M1 proteins of the NS1 R38A/K41A virus could not be detected after 3 passages by western blotting in A549 cells, while the NS1 K41A and R38A viruses showed protein expression levels intermediate between those of the WT and NS1 R38A/K41A viruses (Figure 3B). Consistently, the F2 titer of NS1 R38A/K41A virus was much lower than that of the WT virus and other NS1 mutant viruses and could not be detected in A549 cells after 3 passages by the plaque assay (Table 1 and Figure 3C). These results indicated that the replication of the NS1 R38A/K41A virus was limited compared to that of the WT and other NS1 mutant viruses. NS1 R38A/K41A virus did not possess the ability to reproduce in IFN-competent A549 and MDCK cells after 3 or 4 passages.

**Table 1:** Virus titers of the first (F1) to the fourth passages (F4) of the WT and NS1 mutant viruses in A549 cells.

**Mean titers (log10 PFU/mL) ±SD**	**F1**	**→ (MOI = 0.001)**	**F2**	**F3**	**F4**
WT	5.6 ± 0.1		5.8 ± 0.2	5.5 ± 0.2	5.3 ± 0.1
R38A	4.1 ± 0.1		3.7 ± 0.1	3.4 ± 0.1	3.3 ± 0.2
K41A	4.5 ± 0.2		4.3 ± 0.1	4.2 ± 0.2	4.0 ± 0.1
R38A/K41A	3.3 ± 0.1		2.2 ± 0.1	ND^a^	ND

a *ND means not detected*.

### The NS1-expressing vero cell line facilitates the propagation of NS1 R38A/K41A virus

**Figure 4:**
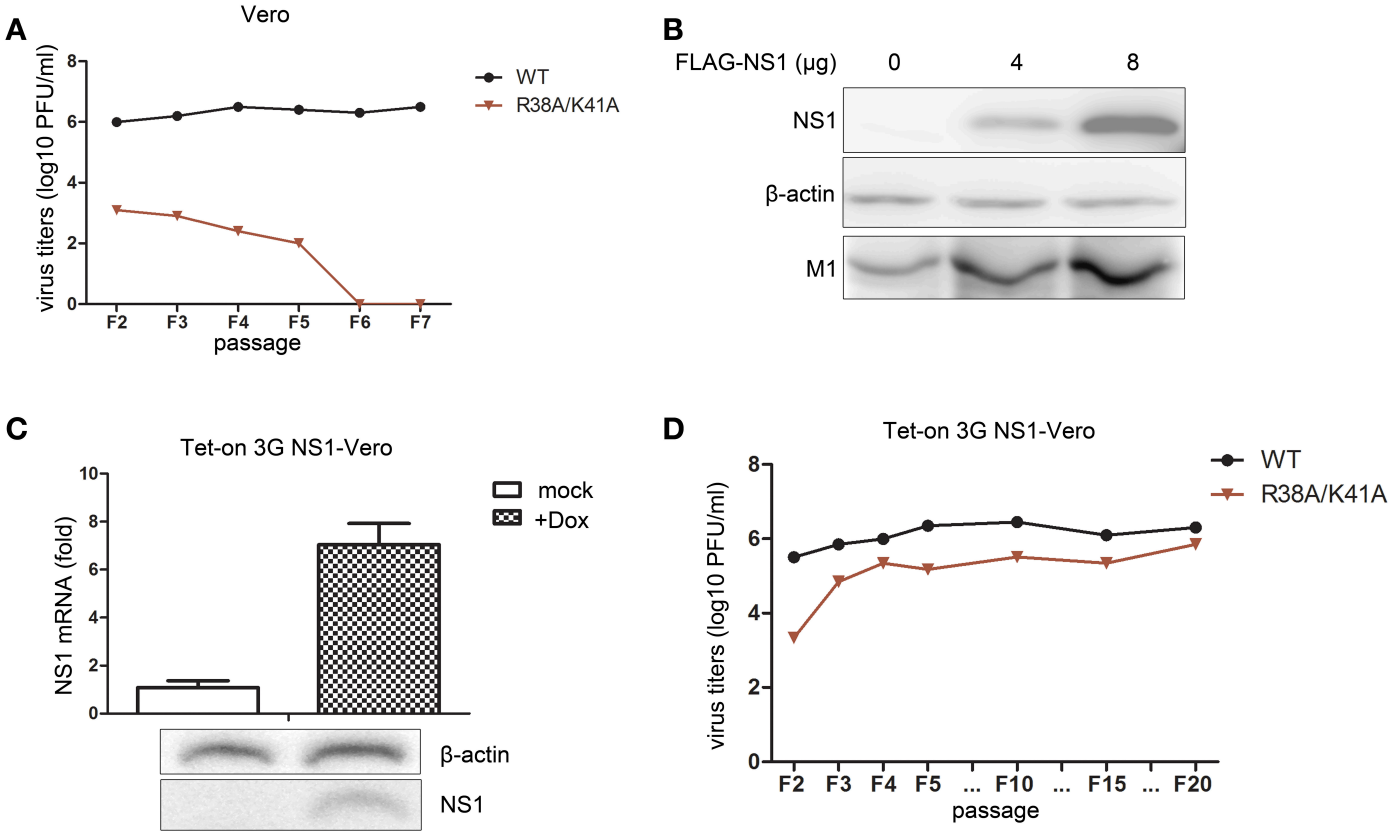
The Tet-On 3G NS1-expressing Vero cell line facilitates the propagation of the NS1 R38A/K41A virus. The rescued WT and NS1 R38A/K41A virus of F1 were blindly passaged in Vero cells. The same volume was used in all infections. Virus titers **(A)** of F2 to F5 WT and NS1 R38A/K41A virus in Vero cells were measured in MDCK cells by plaque assays. The 293T cells were transfected with 0, 4, or 8 μg of FLAG-NS1 plasmid and then infected with NS1 R38A/K41A virus at MOI of 0.01. At 48 h p.t., the cells were lysed for western blot analysis **(B)**. NS1 and M1 were detected with anti-FLAG or M1 antibodies. β-actin was probed as the loading control. Tet-On 3G NS-expressing Vero cells were induced with Dox or were not induced for 24 h. The mRNA **(C**, upper**)** and protein levels **(C**, lower) of NS1 were detected by real-time PCR and western blotting, respectively. The NS1 R38A/K41A and WT viruses from each passage of were obtained from the Tet-On 3G NS1-expressing Vero cells and subjected to plaque assays using MDCK cells to measure the virus titers **(D)**.

We found that the NS1 R38A/K41A virus was an IFN antagonist activity-defective and replication-limited strain that has the potential to become a vaccine candidate, while its replication was inhibited in IFN-competent cells. Thus, we attempted to establish an IFN-deficient cell line to propagate this virus. First, we selected the Vero cell line to propagate this virus, because the gene loci encoding IFN-α/β are missing from the genomic DNA of Vero cells (Emeny and Morgan, 1979). The rescued WT and NS1 R38A/K41A virus of F1 were passaged blindly in Vero cells and subjected to plaque assays on MDCK cells to measure virus titers. The results showed that the titers of the WT and NS1 R38A/K41A viruses in Vero cells (Figure 4A) were a bit little higher than those in A549 cells (Figure 3C). In addition, the WT virus could effectively replicate in Vero cells, while the NS1 R38A/K41A virus could no longer be detected in Vero cells after five passages (Figure 4A), indicating that the absence of IFN-α/β was not enough to help the replication of the NS1 R38A/K41A virus. Then we tried to rescue the functions of NS1 by overexpressing WT NS1 in these cells. The 293T cells were transfected with increasing amounts of WT NS1 and infected with NS1 R38A/K41A virus at 6 h p.t. The expression levels of the NS1 and M1 proteins were then determined by western blotting. The results showed that the exogenous supplementation of WT NS1 protein facilitated the replication of the NS1 R38A/K41A virus (Figure 4B). Therefore, we further established a stable Vero cell line expressing the WT NS1 of WSN virus based on the Tet-On 3G system. NS1 was highly expressed in the Tet-On 3G Vero cells in the presence of Dox (Figure 4C). We next investigated the stability of the NS1 R38A/K41A virus in the Tet-On 3G NS1-expressing Vero cells during successive passaging. The virus titer of each passage of the NS1 R38A/K41A virus from MDCK cells was measured by plaque assays. At the same time, the NS1 genes of F5, F10, F15, and F20 viruses were amplified for sequencing. We found that the NS1 R38A/K41A virus was able to effectively replicated, resulting in titers of 5–6 log10 PFU/mL in the NS1-expressing Vero cells after 3 passages, which was close to those of WT virus (Figure 4D), and the sequences of NS1 from different passages were not changed (data not shown). It was suggested that the NS1 R38A/K41A virus was able to steadily propagate in the IFN-deficient Tet-On 3G NS1 Vero cells for at least 20 passages.

### The replication of the NS1 R38A/K41A virus is limited in mice

**Figure 5:**
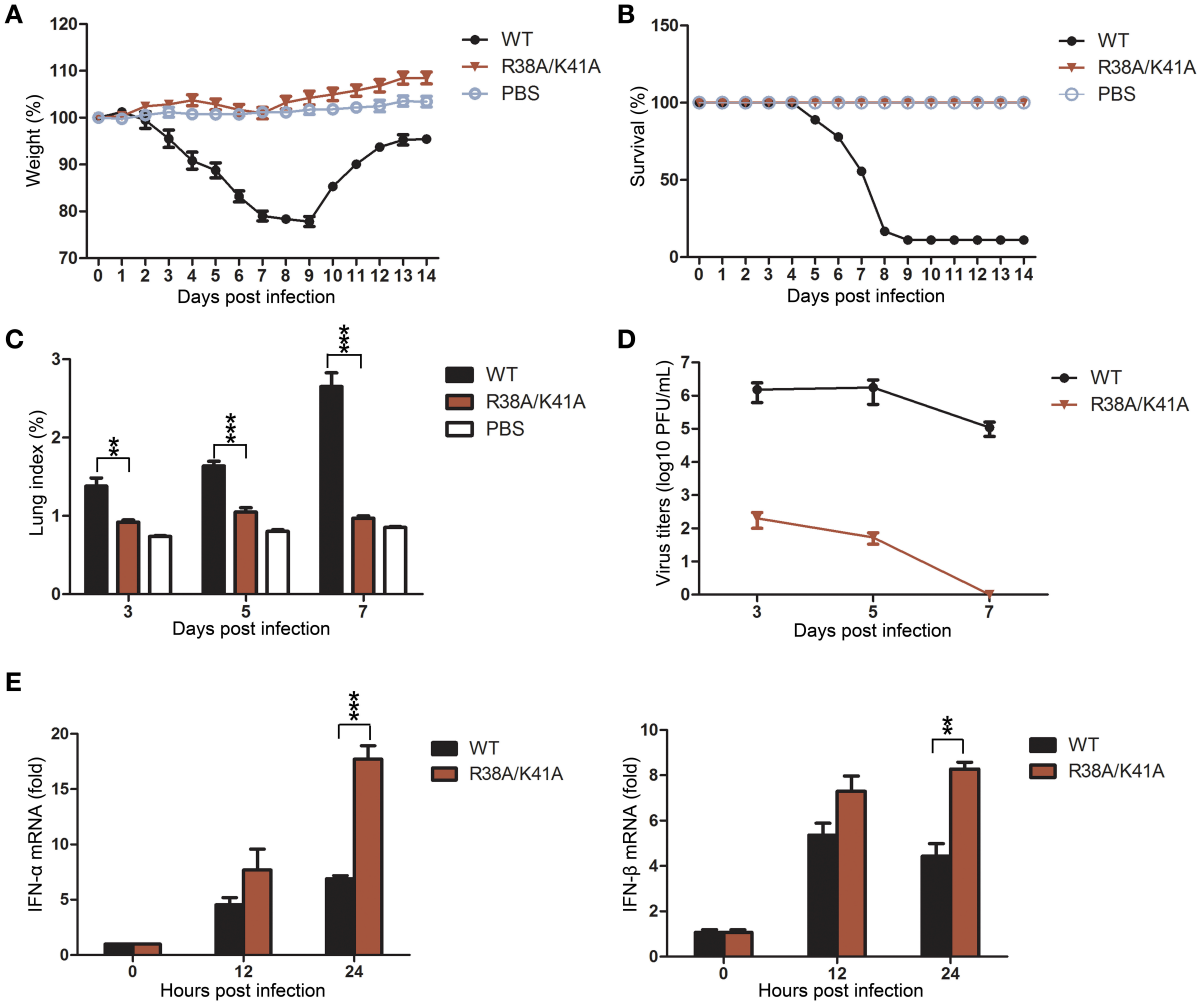
The NS1 R38A/K41A virus exerted full infectivity but its replication is limited in mice. Mice (*n* = 6) were intranasally infected with 10^3^ PFU of the third passage NS1 R38A/K41A virus from the Tet-on NS1-expressing Vero cells, WT virus, or PBS, and then monitored daily for survival for 14 days. The weight curves **(A)** and survival rate **(B)** of mice were determined. Body weights were calculated as the percentage of the original body weight. The data are the mean body weights. The error bars represent the standard deviation of the mean. Three mice infected with different viruses or PBS were sacrificed at the indicated time points, and the lungs were collected. Lung indices **(C)** were calculated and virus titers in lungs **(D)** were determined by plaque assays using MDCK cells. IFN-α and IFN-β mRNA levels in lung tissues **(E)** of mice (*n* = 3) infected with WT or NS1 R38A/K41A virus for 0, 12, and 24 h p.i. were determined by real-time PCR. All data from three independent experiments were analyzed. Data are shown as means with SD. ^**^p < 0.01 and ^***^p < 0.001 (unpaired, two-tailed Student's *t*-test).

To evaluate the replication ability and pathogenicity of the NS1 R38A/K41A virus *in vivo*, 5-week-old female BALB/c mice were intranasally infected with the third passage the NS1 R38A/K41A virus from Tet-on NS1-expressing Vero cells (10^3^ PFU), WT virus (10^3^ PFU), or PBS as a control. The weight and mortality of mice were continuously monitored for 14 days. The weight of mice infected with the WT virus significantly decreased, and the mortality rate was >80%. In contrast, there was no weight loss or death among the NS1 R38A/K41A- or PBS-treated mice (Figures 5A,B).

IAV mainly infects the lungs of the mice, so the lung indices and virus titers in lungs were examined at 3, 5, and 7 days post infection (d p.i.). The lung indices of the NS1 R38A/K41A virus-infected mice were much lower than those infected with the WT virus at 3, 5 and 7 d p.i. (Figure 5C), and the virus titers in lungs of the NS1 R38A/K41A virus-infected mice decreased more than 4 log10 PFU/mL compared with those of WT virus-infected mice at 3 and 5 d p.i. Finally, no virus could be detected in the lungs of NS1 R38A/K41A virus-infected mice at 7 d p.i. (Figure 5D). Moreover, we found that the NS1 R38A/K41A virus triggered robust IFN-α and IFN-β production in mice, while the IFN production in WT virus-infected mice was severely restrained due to the IFN antagonist activity of NS1 (Figure 5E). Collectively, the NS1 R38A/K41A virus exerted full infectivity but was quickly cleared from the lung tissues due to robust innate immune responses.

### The NS1 R38A/K41A virus is immunogenic and protects mice from a lethal challenge

**Figure 6:**
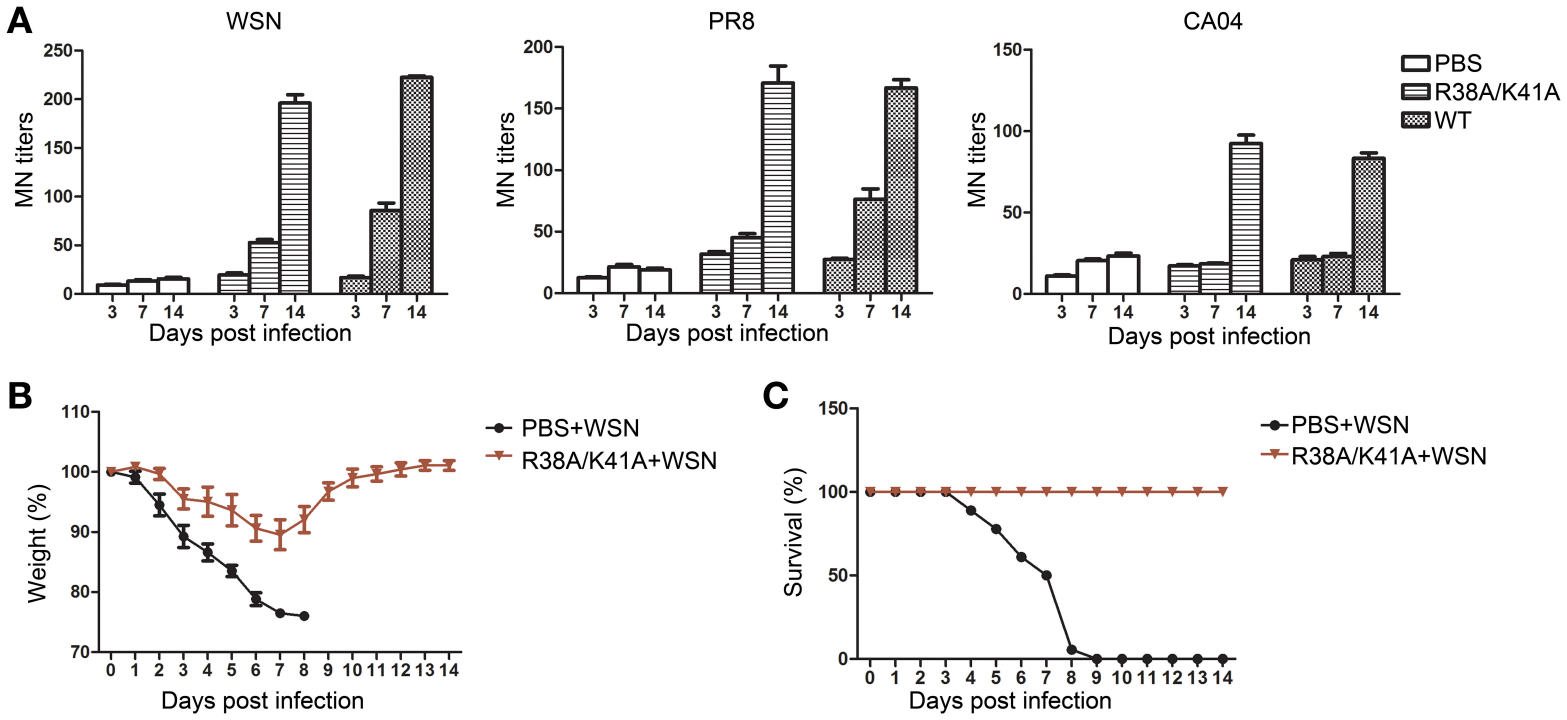
Immunogenicity and protective efficiency of the NS1 R38A/K41A virus. Sera of WT or NS1 R38A/K41A virus infected mice (*n* = 6) was collected at 3, 7, and 14 d p.i. The neutralization titers **(A)** against the WT WSN, A/Puerto Rico/8/1934 (PR8), or A/California/04/2009 (CA04) viruses were determined by MN assays. Mice (*n* = 6) pre-inoculated with PBS or NS1 R38A/K41A virus in the previous experiments were infected with 10^3.88^ PFU of WT WSN virus at 17 d p.i. Body weights **(B)** were examined for another 14 days and calculated as the percentage of the original body weight. The data present are the mean body weights. The survival rate **(C)** of mice (*n* = 6) pre-inoculated with PBS or the NS1 R38A/K41A virus against the WT WSN virus was calculated. All data from three independent experiments were analyzed. Data are shown as means with SD.

To investigate the immunogenicity of the NS1 R38A/K41A virus, sera from the WT or NS1 R38A/K41A virus infected mice was collected at 3, 7, and 14 d p.i. to test the microneutralization (MN) titers against the WT WSN, A/Puerto Rico/8/1934 (PR8) or A/California/04/2009 (CA04) virus. Sera from the NS1 R38A/K41A virus-infected mice at 14 d p.i. showed similar MN titers against WSN, PR8, and CA04 compared with those infected with the WT virus (Figure 6A).

PBS or NS1 R38A/K41A virus-inoculated mice from the previous experiments were infected with WT WSN (10^3.88^PFU) at day 17 after the first infection to test protective efficiency. Body weight and survival were continuously monitored for 14 days. PBS pre-inoculated mice lost weight rapidly and all died at 9 d p.i. (Figures 6B,C). However, the weight of NS1 R38A/K41A virus pre-inoculated mice decreased to a maximum of about 10% at 7 d p.i. and then quickly recovered (Figure 6B). The NS1 R38A/K41A virus pre-inoculated mice all survived against a second infection with WT WSN. In conclusion, the replication-limited IAV vaccine was safe, immunogenic, and protective.

## Discussion

In this study, we demonstrated that the NS1 R38A/K41A virus could not survive in cells and mice for a long time because of its functional defects and that its life cycle was limited. The replication-limited attenuated viruses would be eliminated from the host by the immune system in a relatively short period of time. After several blind passages in A549, MDCK, or Vero cells, the NS1 R38A/K41A virus could hardly be detected. This is a very important feature for a candidate vaccine strain.

The replication-limited and IFN-sensitive influenza viruses, such as NS1-altered or -truncated influenza virus, induce robust type I IFN responses, which, in turn, limit the viral replication. Therefore, this type of virus can only effectively propagate in IFN-deficient systems. Young (6- or 7-day-old) embryonated eggs (Talon et al., 2000b; Chambers et al., 2009) and the Vero cell line (Mosca and Pitha, 1986; Egorov et al., 1998; Chen et al., 2011) are used to grow NS1-deleted virus stocks because young embryonated eggs have an underdeveloped IFN system, and the gene loci encoding IFN-α/β are absent in the genomic DNA of Vero cells (Emeny and Morgan, 1979). But it still remains unknown whether NS1-altered or -truncated influenza virus could be stably passaged in the young embryonated eggs or the Vero cell line. In the present study, we selected the Vero cell line as the production cell line to propagate the NS1 R38A/K41A virus. Although the gene loci encoding type I IFN (IFN-α/β) are absent in Vero cells, those encoding other type of IFNs (such as IFN-λ) are present (Prescott et al., 2010). Our data showed that the NS1 R38A/K41A virus could not be detected in Vero cells after five passages, suggesting that the absence of IFN-α/β was not sufficient for the replication of the NS1 R38A/K41A virus. Furthermore, NS1 is well-known to antagonize the host innate immune responses. Besides the IFN-α/β antagonist activity, NS1 also inhibits RIG-I-mediated IFN-λ production (Onoguchi et al., 2007; Witte et al., 2010), limits the activity of PKR and OAS (Min and Krug, 2006; Min et al., 2007), and participates in the RNAi pathway of the host (Matskevich and Moelling, 2007). Therefore, to further antagonize host innate immune responses, we established a Vero cell line stably expressing the WT NS1 of the WSN virus based on the Tet-On 3G system. The NS1 R38A/K41A virus was able to steadily propagate in this NS1-expressing Vero cell line for at least 20 passages. This was the first presentation of a NS1-expressing Vero cell line, which was in favor of the replication of IFN-sensitive viruses. In our studies, the titers of the NS1 R38A/K41A virus reached 5–6 log10 PFU/mL in the NS1-expressing Vero cells after three passages. More efforts will be undertaken to optimize the manufacturing process in the future.

As previously reported, passaging the NS1 R38A/K41A virus in MDCK cells three times resulted in the selection of a mutant virus containing a third mutation at amino acid residue 42 of the NS1 protein (S42G) (Donelan et al., 2003). However, we did not find the S42G mutation or any other mutation when the NS1 R38A/K41A virus was continuously passaged in NS1-expressing Vero cells for 20 passages. Therefore, the NS1-expressing Vero cell line is a promising IFN-deficient system to propagate the IFN-sensitive influenza vaccine virus.

In summary, we observed that the IFN-sensitive NS1 R38A/K41A influenza virus exerted full infectivity but its replication was limited. Hence, the virus was eliminated from the host by the robust antiviral immune responses in a relatively short period of time. Therefore, we developed a RIG-I-knockout 293T cell line to package the IFN-sensitive virus and a NS1-expressing Vero cell line to propagate the IFN-sensitive virus. This IFN-deficient system promoted the propagation of the IFN-sensitive vaccine virus. This work will provide more insights for the efficient manufacture of IFN-sensitive vaccine viruses.

## Author contributions

LS and WL: supervised the project, designed the study, analyzed the data, and wrote the manuscript; CC and LS: was responsible for planning and conducting the experimental work; WF, JL, WZ, and SZ: provided the technical support; LY and DL: provided materials and analyzed the data.

### Conflict of interest statement

The authors declare that the research was conducted in the absence of any commercial or financial relationships that could be construed as a potential conflict of interest.
